# Comparative Analysis of Porous Alkali-Activated Composites Modified with Commercial and Laboratory-Prepared Phase Change Materials

**DOI:** 10.3390/ma19132864

**Published:** 2026-07-04

**Authors:** Agnieszka Przybek, Michał Łach

**Affiliations:** 1 Faculty of Material Engineering and Physics, Cracow University of Technology, Jana Pawła II 37, 31-864 Cracow, Poland; 2 Interdisciplinary Center for Circular Economy, Cracow University of Technology, Warszawska 24, 31-155 Cracow, Poland

**Keywords:** alkali-activated materials, phase change materials, porosity, energy-efficient construction, thermal regulation

## Abstract

This study presents a comparative evaluation of geopolymer foams incorporating either commercially available shape-stabilized phase change materials (PCMs) or laboratory-developed diatomite–paraffin PCM granules with controlled particle size fractions ranging from <1.6 mm to >2.5 mm. All PCM variants were incorporated at a constant dosage of 7.5 wt.% to isolate the influence of PCM type on the properties of the resulting composites. The commercial materials comprised PX-4, PX15, and PX20 (Rubitherm Technologies GmbH), whereas the laboratory-developed PCM consisted of paraffin immobilized within a porous diatomite matrix to produce granular shape-stabilized composites. The experimental program included the determination of bulk density, total porosity, pore size distribution, thermal conductivity (λ), thermal resistance (R), specific heat capacity (Cp), and compressive strength. The pore structure was characterized by mercury intrusion porosimetry (MIP), while the morphology and dispersion of PCM particles within the geopolymer matrix were investigated using scanning electron microscopy (SEM). All mixtures were produced using the same alkali-activated matrix and identical curing conditions, with the PCM content maintained at 7.5 wt.%. The results demonstrated that the type of PCM significantly affected the microstructure and thermophysical performance of the geopolymer foams. The laboratory-developed diatomite–paraffin PCM provided the most favorable thermal insulation performance, exhibiting the lowest thermal conductivity (0.095 W/m·K) together with the highest thermal resistance (0.278 m^2^·K/W). In contrast, the commercial PX15 and PX20 materials exhibited the highest specific heat capacities (1.740 and 1.778 kJ/kg·K, respectively), indicating superior thermal energy storage capability. In addition, the estimated production cost of the laboratory-developed PCM (2.5–4.0 EUR/kg) was substantially lower than that of the commercial PX materials (approximately 20 EUR/kg), highlighting its potential as a cost-effective alternative for sustainable, energy-efficient building materials. These findings demonstrate that both commercial and laboratory-developed PCM systems can effectively enhance the functionality of geopolymer foams, although they provide different balances between thermal insulation, heat storage capacity, and production cost.

## 1. Introduction

The building sector remains one of the largest consumers of energy worldwide and is responsible for a considerable share of global greenhouse gas emissions. Growing environmental concerns, together with increasingly stringent climate policies, have intensified the search for construction technologies that reduce energy demand while improving the operational performance of buildings. Consequently, considerable research efforts are currently focused on materials and systems capable of limiting heating and cooling loads and enhancing the overall energy efficiency of the built environment [1,2].

Among the technologies currently considered for improving the energy performance of buildings, thermal energy storage (TES) has attracted particular attention. TES systems make it possible to store excess thermal energy during periods of low demand and release it when heating or cooling loads increase. This capability contributes to a more stable indoor thermal environment, reduces peak energy demand, and improves the overall energy efficiency of buildings [3,4].

Thermal energy storage can be achieved through sensible heat, latent heat, or thermochemical storage mechanisms. Among these approaches, latent heat storage based on phase change materials (PCMs) has emerged as one of the most attractive solutions for construction applications. During melting and solidification, PCMs absorb and release substantial amounts of thermal energy while maintaining an almost constant temperature, allowing the heat storage capacity of building components to be significantly increased without markedly increasing their weight [5,6].

Phase change materials are widely recognized as effective functional additives for energy-efficient construction owing to their ability to moderate temperature fluctuations inside buildings. Their incorporation into construction materials reduces the rate of heat transfer through building envelopes, improves thermal inertia, and decreases the energy required for heating and cooling. As a result, PCMs contribute simultaneously to lower operating costs and enhanced thermal comfort for occupants [7].

The increasing application of PCMs is also associated with the rapid expansion of renewable energy technologies. Electricity generated from photovoltaic installations and wind turbines is inherently intermittent, often resulting in a mismatch between energy production and consumption. Thermal energy storage systems incorporating PCMs provide an effective means of utilizing surplus energy by converting it into stored thermal energy that can be recovered during periods of increased demand, thereby improving the flexibility and efficiency of modern energy systems [8].

Despite their considerable advantages, several challenges continue to limit the broader implementation of PCMs in construction materials. The most important issues include leakage during the liquid phase, inherently low thermal conductivity, supercooling phenomena, and the possible deterioration of the mechanical performance of PCM-containing composites. Consequently, intensive research has focused on encapsulation techniques, shape-stabilized PCMs, and the use of porous supporting materials that improve thermal reliability while preventing leakage during repeated phase transitions [9].

Porous construction materials have recently attracted considerable interest as potential carriers for phase change materials. Their interconnected pore network facilitates efficient PCM incorporation, minimizes leakage during repeated melting and solidification cycles, and improves the long-term stability of the composite system. Among the available matrices, alkali-activated materials (AAMs) are regarded as particularly attractive because they combine the ability to incorporate PCMs with a substantially lower environmental impact than conventional Portland cement-based materials. Consequently, the development of porous alkali-activated composites incorporating phase change materials has emerged as a promising strategy for producing sustainable building materials with enhanced thermal energy storage performance and reduced carbon emissions [10,11].

Phase change materials employed for thermal energy storage are generally classified into two categories: commercially available products and laboratory-developed systems designed for specific engineering applications [12,13]. These two groups differ in several important aspects, including manufacturing technology, phase transition temperature range, thermal reliability, production cost, and the possibility of tailoring their physicochemical characteristics to meet particular performance requirements [14,15,16,17,18]. As interest in energy-efficient building technologies continues to increase, both commercial and laboratory-prepared PCMs have become the subject of extensive research aimed at evaluating their effectiveness and applicability in construction materials and thermal energy storage systems [19,20].

Commercial PCMs are generally manufactured as ready-to-use products by specialized companies under strictly controlled production conditions. Their major advantages include stable and reproducible thermophysical properties, high chemical purity, and direct applicability in construction materials without additional processing or modification [21,22,23,24,25]. Among the commercially available PCMs, paraffin waxes, salt hydrates, and eutectic mixtures are the most frequently employed in building applications. These materials typically exhibit phase transition temperatures within the thermal comfort range of buildings, generally between 18 and 30 °C, making them well-suited for passive thermal energy storage and indoor temperature regulation [26,27,28,29].

One of the principal advantages of commercially available PCMs is their excellent thermal and chemical durability. Numerous studies have demonstrated that these materials are capable of maintaining more than 90% of their latent heat storage capacity even after thousands of repeated melting and solidification cycles, making them suitable for long-term use in building applications [30,31,32,33,34]. In addition, commercial PCMs are produced under standardized manufacturing conditions and are subjected to rigorous quality control procedures. As a result, their thermophysical properties are well characterized and highly reproducible, providing designers with reliable performance data for the development of thermal energy storage composites and energy-efficient building materials [35,36].

Despite their well-established advantages, commercially available PCMs are associated with several practical limitations. One of the major drawbacks is their relatively high market price, particularly for microencapsulated products and materials exhibiting high latent heat storage capacity [37,38]. In addition, commercial formulations are supplied with predefined compositions and phase transition temperatures, offering only limited flexibility for further modification. This restricts the possibility of tailoring their thermal characteristics to specific climatic conditions, operating temperatures, or the requirements of individual building applications [39].

To overcome these limitations, increasing research efforts have been directed toward the development of laboratory-prepared PCMs. Unlike commercial products, these materials can be engineered to achieve targeted thermophysical properties and adapted to the requirements of specific applications. They are commonly synthesized by combining fatty acids, fatty alcohols, paraffin waxes, or salt hydrates to form eutectic systems with controlled compositions. By adjusting the proportions of individual components, it is possible to accurately tailor both the phase transition temperature and the latent heat storage capacity of the resulting PCM [40,41,42].

An important advantage of laboratory-developed PCMs is the possibility of incorporating renewable resources and waste-derived feedstocks into their production. Recent studies have increasingly focused on PCMs synthesized from vegetable oils, bio-based fatty acids, waste cooking oils, animal fats, and various by-products of the food processing industry. The use of these alternative raw materials not only lowers production costs but also improves the environmental sustainability of thermal energy storage systems by promoting resource recovery and reducing dependence on fossil-based materials [43,44,45].

In recent years, considerable research has focused on enhancing the thermal conductivity of laboratory-developed PCMs, as their inherently low thermal conductivity restricts the rates of heat storage and release. To overcome this limitation, a wide range of thermally conductive additives has been investigated, including graphene, expanded graphite, carbon nanotubes, graphene oxide, and biochar. The incorporation of these nanostructured materials has been shown to significantly improve heat transfer, in many cases increasing thermal conductivity severalfold while preserving a high latent heat storage capacity and maintaining the phase-change performance of the PCM [46,47,48,49].

At the same time, significant progress has been made in the development of shape-stabilized PCM systems, in which the phase change material is physically confined within the porous framework of a supporting matrix. Various inorganic and carbon-based carriers, including silica aerogels, biochar, expanded graphite, expanded perlite, vermiculite, and polymeric matrices, have been successfully employed for this purpose. Immobilization within these porous supports effectively suppresses PCM leakage during the melting process while enabling the direct incorporation of thermal energy storage materials into cementitious and other construction composites [50,51].

Although substantial progress has been achieved in the development of laboratory-prepared PCMs, several issues still limit their widespread practical application. The most important challenges include ensuring long-term thermal and chemical stability, achieving consistent material quality through reproducible manufacturing procedures, and validating their performance under service conditions representative of real building operation. In contrast to commercially available products, many newly developed PCM systems remain at the experimental stage and have not yet been comprehensively evaluated with respect to long-term durability, cyclic stability, and large-scale implementation [52,53,54].

A survey of the available literature shows that the majority of published studies have concentrated on the characterization of individual PCMs or on the development and optimization of encapsulation and stabilization techniques. In contrast, relatively few investigations have provided a direct comparison between commercially available and laboratory-prepared PCMs evaluated under identical experimental conditions using the same host material. This research gap is particularly apparent for porous alkali-activated materials, whose interconnected pore network makes them attractive candidates for PCM incorporation. Consequently, further studies are required to clarify how the type of PCM influences the thermal, physical, and mechanical performance of alkali-activated composites intended for thermal energy storage applications.

Although numerous studies have investigated the incorporation of phase change materials (PCMs) into construction materials, existing research has primarily focused on individual PCM systems or on different immobilization and encapsulation techniques for cementitious and geopolymer matrices. Commercial PCMs have received particular attention because of their well-established thermophysical properties, consistent quality, and long-term operational stability. In parallel, increasing efforts have been devoted to the development of laboratory-prepared PCMs based on natural porous carriers, such as diatomite, which offer the potential to reduce production costs while increasing the use of naturally occurring and locally available raw materials. Despite these advances, comprehensive comparative studies evaluating commercial and laboratory-developed PCMs under identical experimental conditions and within the same alkali-activated matrix remain scarce. Previous investigations have mainly addressed latent heat storage capacity, thermal stability, or the physicochemical characteristics of individual PCM systems, whereas comparatively little attention has been given to their influence on the physical, mechanical, and thermophysical properties of porous alkali-activated composites. In particular, there is a lack of direct comparisons between commercially available Rubitherm PCMs and laboratory-prepared diatomite–paraffin composites used under the same manufacturing conditions. Therefore, the present study aims to address this research gap by systematically comparing geopolymer foams containing commercial bound PCMs (PX-4, PX15, and PX20) with foamed composites incorporating laboratory-prepared diatomite–paraffin PCM granules. Particular emphasis is placed on evaluating the influence of PCM type on the pore structure, bulk density, compressive strength, thermal conductivity, thermal resistance, specific heat capacity, and microstructure of the resulting composites. Such a comparative approach provides valuable information on the feasibility of replacing commercial PCMs with laboratory-developed diatomite-based materials in sustainable thermal energy storage systems for energy-efficient buildings.

## 2. Materials and Methods

### 2.1. Components of the Geopolymer Matrix and Functional Additives

Class F fly ash obtained from the Skawina Combined Heat and Power Plant (CEZ Skawina S.A., Skawina, Poland) was used as the primary aluminosilicate precursor for the preparation of the geopolymer matrix. Owing to its high content of reactive amorphous phases and a favorable SiO_2_-to-Al_2_O_3_ ratio, the fly ash provides suitable conditions for geopolymerization, resulting in the formation of a stable three-dimensional aluminosilicate network with good mechanical integrity. To improve the dimensional stability of the composites and reduce shrinkage during curing, fine quartz sand supplied by the Świętochłowice Sand Pit (Świętochłowice, Poland) was incorporated as an inert mineral aggregate. Its high purity and controlled particle size distribution contributed to improved packing density and enhanced the structural stability of the geopolymer foams. In addition, Górkal 70 high-alumina cement (Górka Cement Sp. z o.o., Trzebinia, Poland) was introduced as a hydraulic component to accelerate setting and support the early development of mechanical strength. Lightweight fly ash microspheres (TERMO-REX S.A., Jaworzno, Poland) were used to decrease the density of the composites and improve their thermal insulation performance. Their hollow spherical morphology, low density, and relatively high mechanical resistance enabled the production of lightweight geopolymer foams while maintaining satisfactory structural properties. Furthermore, the microspheres promoted a more uniform distribution of pores throughout the material, contributing to the development of a homogeneous porous structure. The foaming process was stabilized by the addition of syringaldehyde (Merck, Darmstadt, Germany), which acted as a surfactant. Its presence facilitated the generation and stabilization of gas bubbles during foaming while reducing their coalescence in the early stages of setting. Consequently, geopolymer foams with a more uniform pore distribution and a higher proportion of closed pores were obtained, contributing to improved thermal insulation performance and favorable mechanical properties.

### 2.2. Characteristics of Commercial and Laboratory-Prepared Phase Change Materials (PCMs)

Four different phase change materials were investigated in this study, including one laboratory-prepared PCM and three commercially available products. The laboratory-developed material consisted of a diatomite–paraffin composite produced by vacuum impregnation of porous diatomite with Flexol paraffin oil using the procedure described in the authors’ previous publications [55,56]. Owing to its highly porous structure, diatomite served as a natural supporting matrix that effectively immobilized the paraffin within its pore network, thereby reducing the risk of leakage during repeated phase transitions. The laboratory-prepared PCM exhibited a flash point of approximately 260 °C and a phase transition temperature range between approximately −30 and −3 °C. Its thermophysical properties varied slightly with the particle size of the granules. The thermal conductivity ranged from 0.1056 to 0.1207 W/(m·K), with the lowest value recorded for the 1.8–2.0 mm fraction and the highest for particles smaller than 1.6 mm. The specific heat capacity varied between 1.16 and 1.55 kJ/(kg·K), with the highest values measured for the >2.5 mm and 1.8–2.0 mm fractions. A detailed description of the preparation method together with the physicochemical characterization of the laboratory-developed PCM has been reported in the authors’ earlier studies [55,56].

The commercially available phase change materials investigated in this study were PX-4, PX15, and PX20, manufactured by Rubitherm Technologies GmbH (Berlin, Germany) as customized powdered PCM products based on the RT series. These materials belong to the group of shape-stabilized PCMs, in which the phase change material is permanently immobilized within an inorganic supporting matrix. This stabilization approach preserves the free-flowing nature of the powder during repeated melting and solidification cycles, enabling its direct incorporation into cementitious and geopolymer composites without additional encapsulation. The three commercial PCMs differed mainly in their phase transition temperatures and thermophysical characteristics, reflecting differences in PCM composition and the proprietary stabilization technology employed by the manufacturer. In contrast, the laboratory-developed material utilized natural diatomite as the supporting matrix for paraffin immobilization. The different carrier materials and stabilization methods may influence not only the thermal energy storage performance but also the interaction between the PCM particles and the geopolymer matrix, thereby affecting the final properties of the composites. The Rubitherm materials were designed for applications covering different operating temperature ranges encountered in energy-efficient buildings. By comparison, the laboratory-prepared diatomite–Flexol composite represents a sustainable alternative based on a naturally occurring mineral carrier, offering the potential for lower production costs and increased utilization of natural raw materials in thermal energy storage composites. The principal thermophysical properties of all investigated PCMs, including the phase transition temperature range, thermal conductivity, specific heat capacity, and flash point, are summarized in Table 1.

The commercial PX-4, PX15, and PX20 materials are powdered shape-stabilized PCMs in which a paraffin-based phase change material is physically retained within an inorganic porous support having an average particle size of approximately 200 μm. The supporting matrix acts as a stabilizing medium, effectively suppressing PCM leakage during repeated melting and solidification cycles while reducing volume changes associated with the phase transition [57]. Both the laboratory-prepared diatomite–Flexol composite and the commercial PX materials belong to the category of shape-stabilized PCMs employing porous mineral carriers for paraffin immobilization. The principal difference between these systems lies in the type of supporting matrix. In the laboratory-developed PCM, naturally occurring diatomite serves as the porous carrier, whereas the commercial PX products utilize a proprietary inorganic adsorbent whose composition has not been disclosed by the manufacturer. Differences in the carrier material and stabilization approach may influence the interaction between the PCM particles and the geopolymer matrix, as well as the thermophysical performance of the resulting composites.

### 2.3. Preparation of Geopolymer Foams Modified with Phase Change Materials (PCM)

Geopolymer foam specimens were prepared using an M/LMB-s laboratory mixer (GEOLAB, Warsaw, Poland). The manufacturing procedure consisted of four main stages: homogenization of the dry constituents, preparation of the alkaline activator, foaming of the fresh mixture, and curing of the hardened composites. Initially, the dry ingredients, including Class F fly ash, the selected phase change material (PCM), and the remaining mineral components described in Section 2.1, were introduced into the mixer and blended for 5 min at 50 rpm to ensure homogeneous distribution throughout the mixture. Subsequently, the alkaline activator was prepared from a 10 M sodium hydroxide (NaOH) solution, produced by dissolving granulated NaOH (purity >99%, PCC Rokita S.A., Brzeg Dolny, Poland) in distilled water, and R-145 sodium silicate (Zakłady Chemiczne ANSER, Wiskitki, Poland) with a SiO_2_/Na_2_O molar ratio of 2.5. The two components were mixed at a mass ratio of 1:2.5, producing the activating solution used for geopolymer synthesis. After the activator was added to the dry mixture, mixing was continued for 10 min, allowing complete wetting of the precursor particles and uniform dispersion of the liquid phase to obtain a homogeneous geopolymer slurry. The porous structure was generated by introducing a 35 wt.% hydrogen peroxide (H_2_O_2_) solution (Hurtownia chemiczna - Distripark.com, Brzeg Dolny, Poland.) as the foaming agent. Oxygen released during the decomposition of H_2_O_2_ under highly alkaline conditions produced an interconnected pore system within the fresh geopolymer matrix. Following the addition of the foaming agent, mixing was continued for an additional 2 min to ensure its uniform distribution and controlled pore formation. The fresh mixtures were immediately cast into laboratory molds lined with protective foil and allowed to expand freely. The specimens were then cured in an SLW 750 laboratory chamber (POL-EKO Aparatura, Wodzisław Śląski, Poland) at 60 °C for 24 h, promoting geopolymerization and stabilization of the cellular structure. After thermal curing, the samples were demolded and stored under laboratory conditions (20 ± 2 °C and approximately 50% relative humidity) for 28 days to allow further development of the geopolymer network and stabilization of the material properties. All specimens were produced as plates measuring 200 × 200 × 25 mm. The designation of each experimental series, together with the detailed mixture compositions, is presented in Table 2.

The phase change material content was set at 7.5 wt.% to allow for a direct comparison of the effect of the type of PCM used while maintaining an identical additive content in all composites. This value was also chosen based on the authors’ previous studies, in which PCM contents of 5 and 10 wt.% were analyzed [56]. The results showed that an increase in the PCM content improves thermal energy storage properties; however, it may also lead to changes in the microstructure and a deterioration of certain mechanical parameters. Conversely, lower PCM contents limit the thermal energy storage effect. Therefore, 7.5 wt.% was adopted as an intermediate level, allowing for a balance between thermal and mechanical properties and ensuring an objective comparison of laboratory-grade and commercial phase change materials. It should be emphasized that the aim of this study was not to optimize the amount of PCM, but to assess the influence of the type of phase change material on the properties of geopolymer foams. The analysis of the influence of different PCM contents is the subject of the authors’ previous research and will be further developed in subsequent studies, taking commercial materials into account.

**Figure 1:**
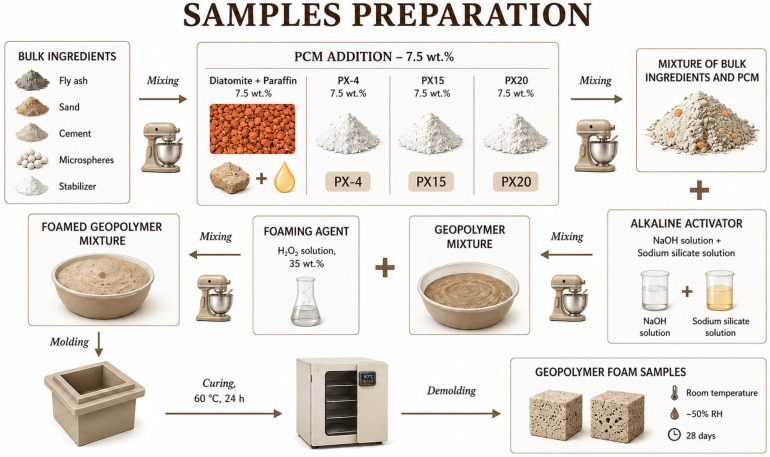
Flowchart of the process for preparing geopolymer foams modified with phase change materials (PCM).

A schematic overview of the sample preparation process, covering the stages of mixing the solid components, dosing the PCMs, alkaline activation, foaming, molding, and curing of the composites, is shown in Figure 1.

### 2.4. Analysis of the Porosity of Geopolymer Composites Containing PCM

The pore structure of the geopolymer foams was investigated using a PoreMaster 33 mercury intrusion porosimeter (Anton Paar, Graz, Austria). This technique enables the characterization of porous materials over a broad pore size range by measuring the pressure required for mercury to penetrate the pore network. During the analysis, mercury is progressively forced into the pores under increasing pressure, allowing the determination of key structural parameters, including total porosity, pore size distribution, total intruded pore volume, and specific surface area. The porosimeter operates over a wide pressure range (0.2–50 psi in the low-pressure stage), making it suitable for the characterization of both fine and coarse pores commonly found in porous geopolymer materials. Mercury intrusion porosimetry (MIP) is a well-established method for evaluating the internal structure of cementitious and alkali-activated materials and provides valuable information on the connectivity and accessibility of the pore system. Measurements were performed for all geopolymer foam formulations prepared in this study. The obtained results were used to evaluate the influence of the different phase change materials on pore structure development and to compare changes in total porosity, pore size distribution, total intruded mercury volume, and specific surface area among the investigated composites.

### 2.5. Compressive Strength of Geopolymer Composites Containing PCM

The compressive strength of the produced geopolymer foams was determined using an MTS Criterion 43 universal testing machine (MTS Systems Corporation, Eden Prairie, MN, USA) controlled by TestSuite 1.0 software. The testing system operates with a maximum load capacity of 30 kN and enables continuous acquisition of load and displacement data throughout the measurement. Compression tests were performed in accordance with the requirements of EN 196-1: Test Methods for Cement—Part 1: Determination of Strength [58]. Cubic specimens with dimensions of 25 × 25 × 25 mm were prepared for mechanical testing. Before loading, each specimen was carefully positioned at the center of the compression platens to ensure uniform stress distribution during the test. The load was then applied continuously until complete failure of the specimen occurred. The maximum failure load (Fc) recorded during each measurement was used to calculate the compressive strength (Rc) according to Equation (1).

(1) $$Rc=\frac{F_{c}}{A}[MPa]$$

where

Rc—compressive strength [MPa],
*A*—sample cross-sectional area [mm^2^],
*F_c_*—maximum load [N].

However, attention should be paid to the limitations associated with the mechanical testing methodology used. Compressive strength was determined on specimens measuring 25 × 25 × 25 mm, in accordance with the established testing procedure for geopolymer materials of limited dimensions. In the case of materials with a highly developed porous structure and containing PCM granules of varying sizes—especially those exceeding 2.5 mm—the possibility of a scale effect cannot be ruled out. The presence of individual larger granules and local structural inhomogeneities may lead to stress concentrations and increased variation in strength results. For this reason, the obtained values should be interpreted primarily as comparative results between the analyzed material variants, since all specimens were prepared according to an identical manufacturing procedure, had the same dimensions, and were tested under the same conditions. In future studies, it will be appropriate to conduct additional tests using larger specimens, which will allow for an assessment of the size effect on mechanical properties and increase the representativeness of the obtained results.

### 2.6. Thermal Properties of Geopolymer Composites Containing PCM

The thermophysical properties of the geopolymer foams were evaluated using an HFM 446 Lambda heat flow meter (Netzsch, Selb, Germany). The instrument operates according to the Heat Flow Meter (HFM) method, in which heat is transferred through the specimen positioned between two plates maintained at different temperatures. This technique is widely applied for the characterization of thermal insulation and building materials and complies with the requirements of ASTM C1784 [59], ASTM C518 [60], ISO 8301 [61], and EN 12664 [62]. The HFM 446 Lambda system is capable of measuring thermal conductivity within the range of 0.007–2.0 W/(m·K), making it suitable for both lightweight insulating materials and denser construction composites. Stable testing conditions were ensured by Peltier-controlled measuring plates, which provided precise temperature regulation and high measurement repeatability. The thermal conductivity (λ) and thermal resistance (R) of the geopolymer foams were determined within the temperature range of 0–20 °C, corresponding to the typical operating conditions of building insulation materials. In addition, the specific heat capacity (Cp) was measured between 27.5 and 32.5 °C, a temperature interval selected to evaluate the heat storage capability of the investigated composites under conditions representative of indoor building environments. Plate specimens matching the dimensions of the measurement chamber were used for all thermophysical measurements. Before testing, the mass of each specimen was determined using a RADWAG PS 200/2000.R2 analytical balance (Radom, Poland) with an accuracy of 0.01 g, while the specimen dimensions were measured with a digital caliper having a resolution of 0.01 mm. These measurements were used to calculate the bulk density of the composites. Based on the experimental data, the thermal conductivity, thermal resistance, specific heat capacity, and bulk density of all geopolymer foam variants were determined and subsequently compared.

### 2.7. Macroscopic Photographs of Geopolymer Composites Containing PCM

The surface morphology and macroscopic pore structure of the geopolymer foams were examined using a VHX-7000 digital optical microscope (Keyence, Mechelen, Belgium). Before observation, the specimen surfaces were carefully cleaned and gently leveled to remove loose particles and improve image quality. This preparation ensured reliable visualization of the surface features without altering the original morphology of the material. Macroscopic images were acquired in reflected light using the microscope’s integrated ring illumination system and automatic focusing function, allowing accurate imaging of the porous structure without additional specimen preparation. To improve image quality and enhance the visibility of surface features, the High Dynamic Range (HDR) mode was applied, providing optimized exposure and increased contrast. In addition, the Depth Composition function was used to generate fully focused images with an extended depth of field, enabling detailed observation of pore morphology, pore distribution, and the overall homogeneity of the geopolymer foam structure. The obtained macroscopic images were used for the qualitative evaluation of the cellular structure and for comparison of the effects of the different phase change materials on the morphology of the geopolymer foams.

### 2.8. Microscopic Images of Geopolymer Composites Containing PCM

The microstructure of the geopolymer foams was examined using a JEOL IT200 scanning electron microscope (SEM) (JEOL Ltd., Akishima, Tokyo, Japan). SEM observations were performed to evaluate the morphology of the geopolymer matrix, characterize the pore structure, and assess the distribution of the incorporated phase change materials within the composites. Representative fragments were collected from the cured specimens and carefully cleaned to remove loose particles generated during sample preparation. To avoid possible thermal degradation or migration of the phase change materials, the specimens were dried at 40 °C until a constant mass was reached. The dried samples were then mounted on aluminum stubs using conductive carbon tape and EM-Tec C33 carbon adhesive to ensure stable fixation and efficient dissipation of electrical charge during imaging. Because the geopolymer matrix is electrically non-conductive, the specimen surfaces were coated with a thin gold layer using a DII-29030SCTR Smart Coater (JEOL Ltd., Peabody, MA, USA) prior to microscopic examination. SEM observations were carried out at different magnifications to obtain detailed information on the morphology of the geopolymer matrix, the pore architecture, and the interfacial interactions between the PCM particles and the surrounding matrix. The acquired micrographs were subsequently used to compare the influence of the different phase change materials on the microstructural characteristics of the geopolymer foams.

### 2.9. Use of Artificial Intelligence (AI) Tools

During the preparation of this manuscript, the authors used ChatGPT (OpenAI, GPT-5.5) to assist in the generation of the graphical concept and visual layout of Figure 1. The AI-generated output was subsequently reviewed, edited, and verified by the authors to ensure its scientific accuracy and consistency with the manuscript. The authors take full responsibility for the content of this publication.

## 3. Results

### 3.1. Analysis of the Porosity of Geopolymer Composites Containing PCM

Table 3 presents the parameters describing the porous structure of the tested geopolymer foams containing phase change materials (PCMs). The analysis covered all the composite variants developed, which made it possible to assess the influence of the type of PCM used on the development of the material’s pore structure. The table includes the most important parameters used to characterize porous materials. These include total porosity, reflecting the proportion of pore space in the sample volume, as well as the pore size distribution, which allows for an assessment of the degree of microstructural diversity. The specific surface area values are also presented, which are key indicators influencing heat transfer, sorption, and the material’s interaction with its surroundings. Additionally, the table includes results regarding the total volume of mercury injected into the structure during the measurements. This parameter provides information on the available pore volume and the degree of pore development within the geopolymer matrix. Analysis of the obtained data enables a quantitative and qualitative assessment of the changes occurring in the material structure under the influence of the PCM additive.

Based on the results presented in Table 3, it can be concluded that the use of phase change materials significantly affected the pore structure of the geopolymer foams; however, the direction and magnitude of these changes depended on both the PCM type and the particle size of the laboratory-prepared diatomite–paraffin granules. The reference sample (AAM-REF) exhibited a total porosity of 8.37%, a specific surface area of 0.025 m^2^/g, and a total intruded mercury volume of 0.310 cm^3^/g. The incorporation of the laboratory-prepared PCM noticeably modified these parameters. The greatest increase in total porosity was observed for the <1.6 mm fraction, reaching 20.88%, which was more than twice the value obtained for the reference material. This sample also exhibited one of the highest total intruded mercury volumes (0.522 cm^3^/g), indicating a well-developed and readily accessible pore network. The increased porosity may be associated with a more homogeneous distribution of fine PCM granules within the geopolymer matrix, promoting the formation of additional voids during the foaming process. A comparable total intruded mercury volume (0.522 cm^3^/g) was obtained for the composite containing the >2.5 mm fraction, although its total porosity was lower (15.65%). At the same time, this material exhibited the highest specific surface area among all investigated composites (0.080 m^2^/g), suggesting a more developed internal pore surface that may enhance the interaction between the geopolymer matrix and the PCM. Different behavior was observed for the 2.0–2.5 mm fraction, which exhibited the lowest total porosity among all samples containing the laboratory-prepared PCM (6.93%), even lower than that of the reference sample. Nevertheless, its total intruded mercury volume (0.315 cm^3^/g) remained close to that of AAM-REF, indicating a more compact pore structure while maintaining a similar accessible pore volume. Commercial PX materials affected the pore structure differently. The PX-4 sample exhibited a total porosity of 11.16%, representing a moderate increase compared with the reference material, whereas PX15 showed a porosity of 7.17%, close to that of AAM-REF. The most pronounced effect was observed for PX20, which exhibited the lowest total porosity (1.60%), the smallest specific surface area (0.011 m^2^/g), and the lowest total intruded mercury volume (0.084 cm^3^/g). These results indicate a substantial densification of the geopolymer matrix and a significant reduction in the amount of accessible pore space. This behavior may be attributed to the fine mineral carrier incorporated into the commercial PX20 material, which likely promoted more efficient filling of the voids within the geopolymer structure. Regardless of the PCM type, the pore diameter range remained relatively similar, varying approximately between 4 and 240 μm. Therefore, the incorporation of PCM primarily affected the amount and accessibility of pore space rather than fundamentally changing the pore size distribution. Overall, the laboratory-prepared diatomite–paraffin PCM generally increased the porosity and specific surface area of the geopolymer foams, whereas the commercial PX materials, particularly PX20, produced a pronounced densification effect. These structural differences are expected to influence the thermal and mechanical performance of the composites, as discussed in the following sections.

### 3.2. Compressive Strength of Geopolymer Composites Containing PCM

**Figure 2:**
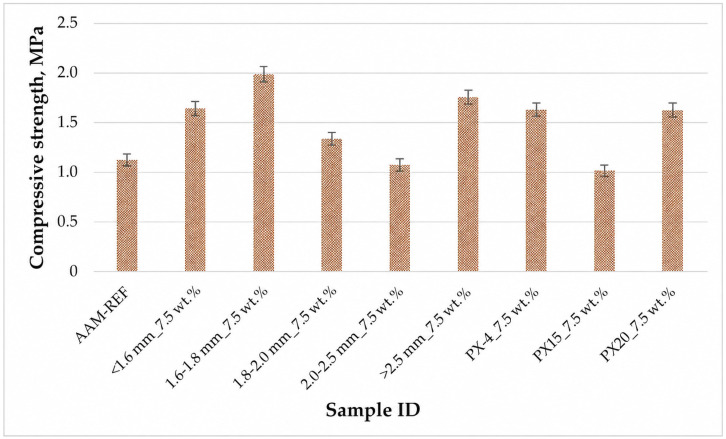
The effect of phase change materials on the compressive strength of geopolymer composites.

The results of the compressive strength tests on the geopolymer foams under investigation are presented in Table 4 and Figure 2. The analysis covered all variants of composites containing phase change materials (PCMs), which made it possible to assess the influence of the type of additive used on the mechanical properties of the resulting materials. Compressive strength is one of the fundamental parameters determining the suitability of geopolymer foams for construction applications, as it defines the material’s ability to bear loads without losing structural integrity. In the case of porous materials, this parameter is particularly important, as it is closely related to the density, porosity, and microstructure of the composite. To ensure the reliability of the results, each test series was subjected to five independent measurements. Based on the results obtained, average values were calculated and are presented in Table 4, whilst their graphical interpretation is shown in Figure 2. This approach made it possible to limit the influence of random measurement deviations and increased the representativeness of the data obtained.

The values obtained for the individual composites ranged from 1.02 to 1.98 MPa, indicating a clear influence of the phase change materials used on the mechanical properties of the foams. The reference sample (AAM-REF), which did not contain PCM, achieved a compressive strength of 1.12 MPa. For most composites containing laboratory-grade PCM based on diatomite and paraffin, higher values of this parameter were recorded. The highest compressive strength was obtained for the sample containing the 1.6–1.8 mm fraction, which reached 1.98 MPa. High values were also observed for samples with a fraction >2.5 mm (1.74 MPa) and <1.6 mm (1.64 MPa). Slightly lower strength values were obtained for composites containing granules of 1.8–2.0 mm and 2.0–2.5 mm in size, for which the strength was 1.34 and 1.08 MPa, respectively. In the latter case, the result obtained was similar to the value recorded for the reference sample. In the group of materials containing commercial PCMs, the highest strength was exhibited by the PX-4 and PX20 samples, reaching 1.62 and 1.60 MPa, respectively. In contrast, the lowest value among all the analyzed composites was recorded for the PX15 sample, for which the compressive strength was 1.02 MPa. The results obtained indicate that the use of phase change materials affects the mechanical properties of geopolymer foams; however, the nature and extent of these changes depend on the type of PCM used.

The observed differences in strength between the individual composites can be attributed not only to the total porosity of the material, but also to the nature of the interaction between the phase change material and the geopolymer matrix. In the case of the laboratory-prepared PCM, paraffin was adsorbed into the porous structure of the diatomite, allowing the material to maintain its shape stability during the phase transition. During the melting of paraffin, a phase transition from the solid to the liquid state occurs, accompanied by a slight change in volume and a local redistribution of stresses within the porous matrix. The well-developed capillary structure of diatomite allows for the compensation of these changes and limits the migration of the liquid phase, reducing the risk of local damage to the geopolymer matrix. At the same time, a suitably developed pore structure can act as stress-relief zones, reducing stress concentration and promoting a more uniform distribution of stresses under mechanical loading. In contrast, in materials with a more compact microstructure containing commercial PX materials, the proportion of voids capable of compensating for local deformations is lower, which may lead to greater stress concentration at the phase boundary between the PCM particles and the geopolymer matrix. It should be emphasized, however, that the mechanisms presented are interpretive in nature and require confirmation through studies involving multiple melting and solidification cycles, as well as an analysis of changes in microstructure and mechanical properties during service.

### 3.3. Thermal Properties of Geopolymer Composites Containing PCM

Table 5 presents the results of tests on the thermophysical properties of geopolymer foams containing various phase change materials (PCMs), including both a laboratory-prepared diatomite–paraffin composite and the commercial materials PX-4, PX15, and PX20. The analysis covered parameters relevant to the use of the materials as thermal insulators capable of storing energy, including the thermal conductivity (λ), thermal resistance (R), specific heat (Cp), and bulk density. The thermal conductivity and thermal resistance were determined in the temperature range of 0–20 °C, corresponding to typical operating conditions for insulation materials used in construction. These parameters enable the assessment of a material’s ability to limit heat flow and the determination of its thermal insulation efficiency. In addition, the specific heat was determined in the temperature range of 27.5–32.5 °C, which allowed the ability of the tested composites to accumulate thermal energy to be assessed. This parameter is particularly important for materials containing PCM, as it influences the amount of energy stored during temperature changes. The analysis also took into account the bulk density of the samples, which is one of the fundamental parameters describing the material’s structure. This value allows for an assessment of the composite’s degree of compaction and constitutes an important characteristic of porous materials intended for thermal insulation applications. All values presented in the table are averages obtained from two independent measurements carried out for each test series. The use of repeated measurements increased the reliability of the results obtained and reduced the impact of random experimental deviations.

The analyzed composites exhibited a bulk density ranging from 353.85 to 450.94 kg/m^3^, confirming their lightweight and porous character. The highest density was recorded for the sample containing laboratory-prepared PCM with a particle size of 1.8–2.0 mm (450.94 kg/m^3^), whereas the lowest value was obtained for the 2.0–2.5 mm fraction (353.85 kg/m^3^). The reference sample (AAM-REF) exhibited a density of 402.62 kg/m^3^. The thermal conductivity coefficient (λ) of the investigated materials ranged from 0.095 to 0.109 W/(m·K). The lowest thermal conductivity (0.095 W/(m·K)) was obtained for the composites containing laboratory-prepared PCM with particle size fractions of <1.6 mm and 2.0–2.5 mm, whereas the highest value (0.109 W/(m·K)) was recorded for the PX20 sample. Compared with the reference material (0.103 W/(m·K)), most composites containing laboratory-prepared PCM exhibited slightly lower thermal conductivity, indicating an improvement in their thermal insulation performance. A similar trend was observed for thermal resistance (R), which ranged from 0.241 to 0.278 m^2^·K/W. The highest thermal resistance (0.278 m^2^·K/W) was achieved by the composite containing the 2.0–2.5 mm fraction, whereas the lowest value (0.241 m^2^·K/W) was recorded for the PX20 sample. The reference material exhibited a thermal resistance of 0.259 m^2^·K/W. Most composites incorporating laboratory-prepared PCM showed thermal resistance values comparable to or higher than those of the reference sample. The greatest differences among the investigated materials were observed for specific heat (Cp), which varied from 1.494 to 1.778 kJ/(kg·K). The lowest value was recorded for the reference sample, whereas the highest was obtained for the PX20 composite. High specific heat values were also measured for PX15 (1.740 kJ/(kg·K)) and PX-4 (1.638 kJ/(kg·K)). Among the composites containing laboratory-prepared PCM, the highest specific heat (1.552 kJ/(kg·K)) was achieved for the >2.5 mm fraction, while the remaining variants exhibited values within a relatively narrow range of 1.512–1.532 kJ/(kg·K). Overall, the results demonstrate that the incorporation of different phase change materials influenced the thermophysical properties of the geopolymer foams to varying degrees. The most pronounced differences were observed in the specific heat capacity, whereas the thermal conductivity and thermal resistance remained within relatively narrow ranges for all investigated composites.

An analysis of the results indicates that the thermal properties of the tested composites were closely related to the characteristics of their porous structure. It should be emphasized that the insulating efficiency of porous materials is determined not only by total porosity, but also by the size, shape, distribution, and degree of interconnection of the pores. In materials with a predominant proportion of fine and closed pores, heat transfer through the gas phase is limited, and the path of heat flow through the solid matrix is lengthened. Consequently, a decrease in the thermal conductivity coefficient and an increase in thermal resistance are observed.

In the conducted studies, samples containing a laboratory-prepared PCM based on diatomite and paraffin were characterized by a more developed porous structure, which resulted in the lowest thermal conductivity values. At the same time, the porous microstructure of diatomite may have increased the number of phase boundaries within the composite, causing additional heat flux dissipation and limiting effective energy transport. In contrast, composites containing PX materials exhibited lower total porosity and a lower volume of intruded pores, indicating a more compact microstructure. This structure promotes the formation of continuous heat conduction paths within the geopolymer matrix, which was reflected in slightly higher values of the λ coefficient. At the same time, these materials achieved the highest specific heat values, indicating a greater proportion of the active phase that stores thermal energy. The results obtained thus indicate that the thermal properties of geopolymer foams are determined not only by the amount of PCM used, but primarily by the interaction between the pore microstructure, the type of phase change material carrier, and the method of its integration into the geopolymer matrix.

### 3.4. Macroscopic Images of Geopolymer Composites Containing PCM

**Figure 3:**
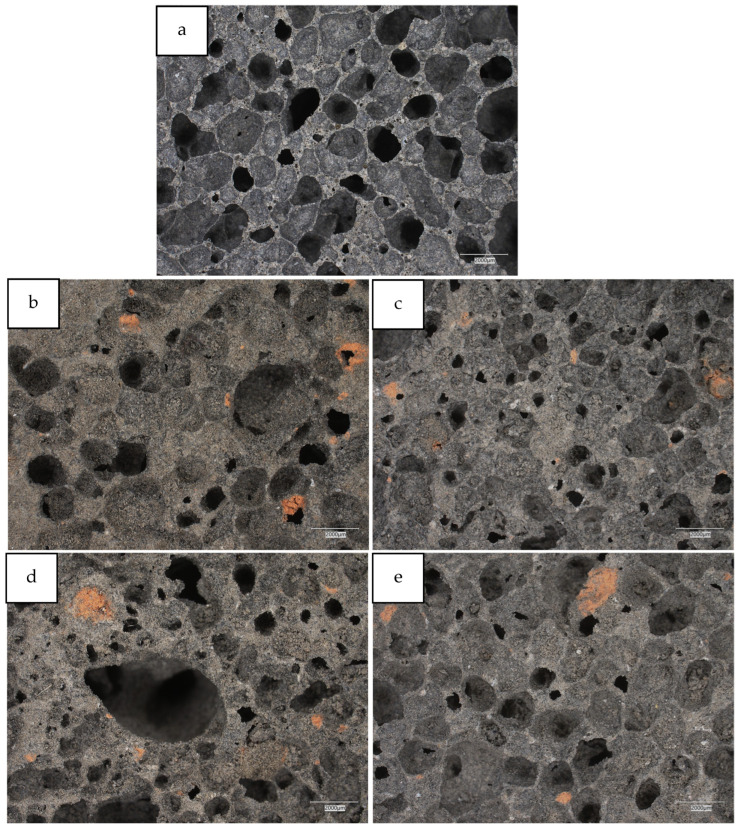

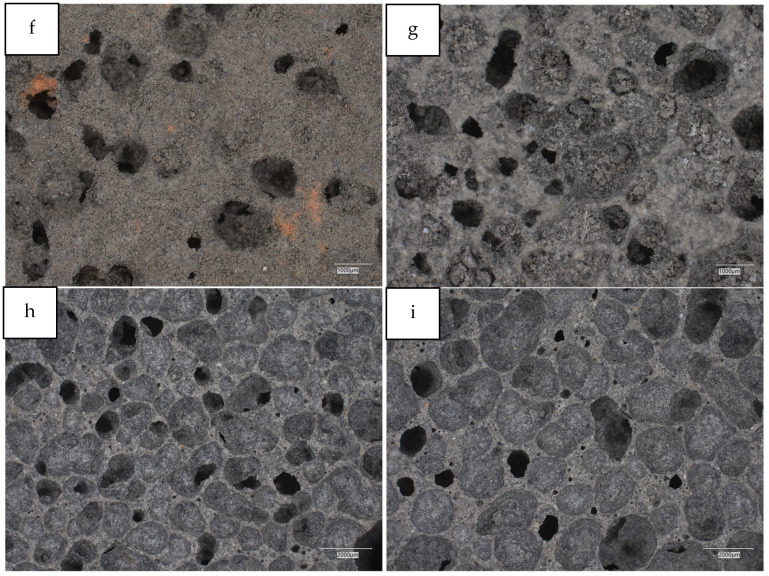
Macroscopic images of geopolymer composites containing PCM: (**a**) AAM-REF, (**b**) <1.6 mm_7.5 wt.%, (**c**) 1.6–1.8 mm_7.5 wt.%, (**d**) 1.8–2.0 mm_7.5 wt.%, (**e**) 2.0–2.5 mm_7.5 wt.%, (**f**) >2.5 mm_7.5 wt.%, (**g**) PX-4_7.5 wt.%, (**h**) PX15_7.5 wt.%, (**i**) PX20_7.5 wt.%.

Figure 3 shows macroscopic images of the geopolymer foams under investigation, obtained using a digital optical microscope. The 20× magnification used enabled a detailed assessment of the sample surfaces and the identification of structural features associated with the presence of phase-changing materials in the geopolymer matrix. The images obtained allow for a qualitative analysis of pore distribution, the degree of structural homogeneity, and visible morphological differences between the individual composite variants. Additionally, it was possible to observe the distribution of PCM particles and their interaction with the surrounding geopolymer matrix. In the case of samples containing a laboratory-prepared diatomite–paraffin composite, grains of varying sizes were observed, whereas for materials containing PCMs from the PX series, more homogeneous surface structures were visible. Macroscopic analysis provides a significant complement to the results obtained by quantitative methods, enabling a visual assessment of the quality of the materials produced and the identification of features that may influence their performance properties. The observations presented in Figure 3 also allow for a preliminary assessment of the influence of the type of PCM used on the development of the porous structure of geopolymer foams.

The macroscopic images shown in Figure 3 indicate that all the composites studied were characterized by a well-developed porous structure typical of geopolymer foams. Regardless of the phase-changing material used, both larger pores of irregular shape and numerous fine voids evenly distributed throughout the matrix volume were observed. The reference sample AAM-REF (Figure 3a) exhibited a relatively homogeneous distribution of pores of varying diameters. The material structure was well-developed, although isolated, and larger voids formed during the foaming process were observed locally. The geopolymer matrix was characterized by a compact structure and a relatively small number of visible inhomogeneities. In the case of composites containing laboratory-grade PCM based on diatomite and paraffin (Figure 3b–f), characteristic light-brown particles corresponding to the grains of the phase change material were visible. Their presence confirms the successful incorporation of PCM into the geopolymer matrix. The distribution of the granules was relatively uniform, although small clusters of particles were observed locally. In samples containing smaller PCM fractions (<1.6 mm and 1.6–1.8 mm), the pore structure appeared more varied, with a greater number of fine voids distributed throughout the volume of the material. As the particle size increased, a more pronounced presence of individual PCM granules and locally larger pores formed in their immediate vicinity was observed. The sample containing the 2.0–2.5 mm fraction (Figure 3e) was characterized by a relatively homogeneous structure and an even distribution of pores. In contrast, for the composite containing granules larger than 2.5 mm (Figure 3f), individual larger PCM particles and a more varied surface morphology were visible. A different picture was obtained for materials containing commercial PCMs PX-4, PX15, and PX20 (Figure 3g–i). In these samples, no clearly distinct phase change material grains were observed, which is due to the different form and nature of the carrier used. The surface structure was more homogeneous, and the pores exhibited a more regular shape and uniform distribution. In particular, in the case of the PX15 and PX20 samples, a compact matrix structure and a smaller number of large voids visible on the material’s surface were noticeable. The images obtained confirm that the type of phase change material used influences the morphology of geopolymer foams. At the same time, all the composites tested retained the multi-porous structure characteristic of geopolymer foams, showing no macroscopic signs of component segregation or defects that might indicate an irregular course of the manufacturing process.

### 3.5. Microscopic Images of Geopolymer Composites Containing PCM

**Figure 4:**
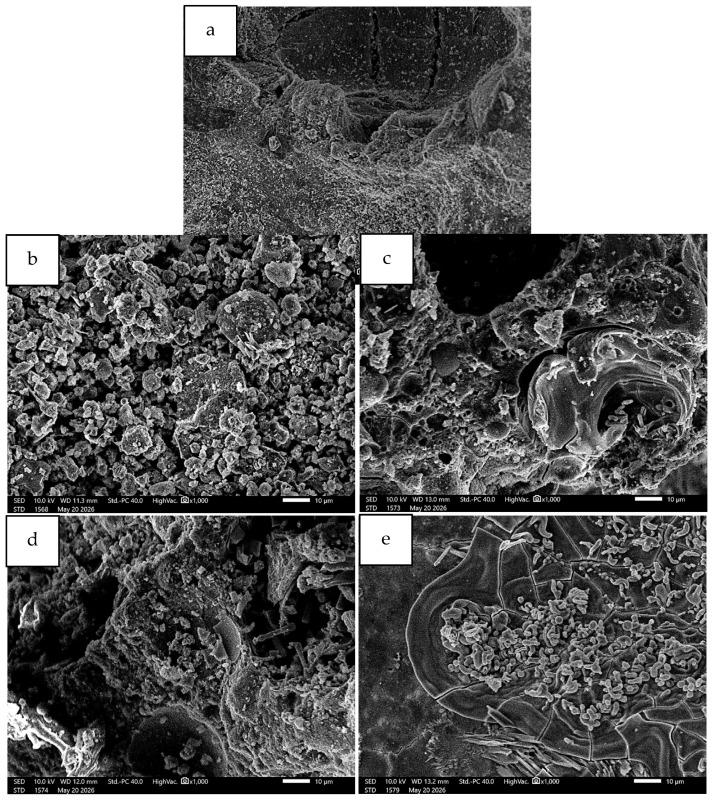

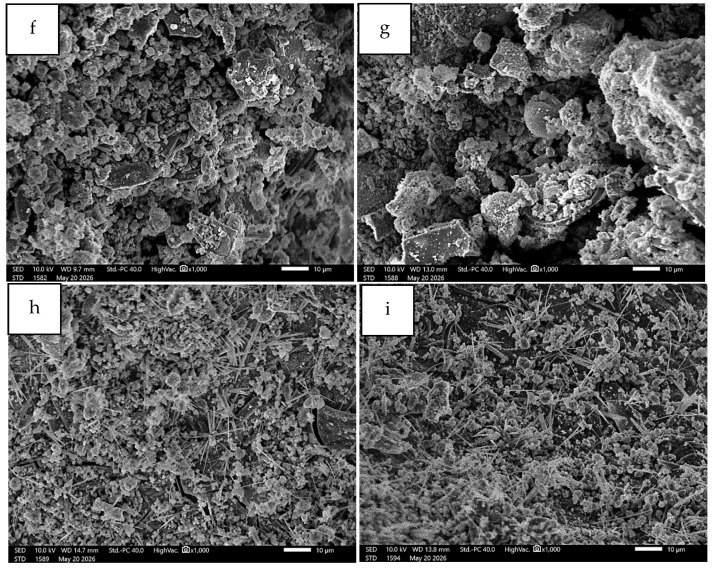
Microscopic images of geopolymer composites containing PCM: (**a**) AAM-REF, (**b**) <1.6 mm_7.5 wt.%, (**c**) 1.6–1.8 mm_7.5 wt.%, (**d**) 1.8–2.0 mm_7.5 wt.%, (**e**) 2.0–2.5 mm_7.5 wt.%, (**f**) >2.5 mm_7.5 wt.%, (**g**) PX-4_7.5 wt.%, (**h**) PX15_7.5 wt.%, (**i**) PX20_7.5 wt.%.

Figure 4 shows images of the microstructure of the geopolymer foams under investigation, obtained using scanning electron microscopy (SEM) at magnifications of 500× and 1000×. The magnifications used enabled a detailed assessment of the geopolymer matrix morphology, pore structure, and the distribution of phase-changing materials within the composites. The micrographs obtained allow for the identification of microstructural features not visible during macroscopic observation, such as local porosity, the presence of microcracks, the degree of matrix compaction, and the nature of the interface between the geopolymer phase and the additive particles. Image analysis also enables the assessment of the material’s homogeneity and the degree of integration of the PCMs used with the geopolymer matrix.

The SEM micrographs shown in Figure 4 confirm the developed and complex microstructure of all the geopolymer foams studied. Regardless of the type of phase-separated material used, a characteristic geopolymer matrix with a heterogeneous morphology and numerous pore spaces was formed as a result of the foaming process. The reference sample AAM-REF (Figure 4a) was characterized by a compact matrix structure with visible fragments of the geopolymer gel phase and local voids resulting from the foaming process. The fracture surface was relatively uniform, with no clearly distinct particles of foreign phases. In the case of samples containing laboratory-prepared PCM based on diatomite and paraffin (Figure 4b–f), numerous irregular particles with a highly developed surface were observed, characteristic of the porous structure of diatomite. Both individual grains and their local agglomerates embedded in the geopolymer matrix were visible. In many places, these particles were well bound to the surrounding geopolymer phase, with no distinct gaps at the phase boundary. This was particularly evident in samples containing the 1.6–1.8 mm and 1.8–2.0 mm fractions, where a uniform distribution of PCM particles within the material structure was observed. In the images of samples containing larger PCM fractions (Figure 4e,f), larger clusters of diatomite grains and more pronounced surface roughness were noticeable. Residues of paraffin layers covering the surface of the mineral matrix were also visible in places, which may indicate an effective process of diatomite impregnation with the phase change material. Microphotographs of composites containing commercial PX materials (Figure 4g–i) showed a different morphology. The structure was more homogeneous, and clearly distinct grains were observed much less frequently than in the case of diatomite-paraffin composites. In the PX15 and PX20 samples, numerous fine, needle-like, and plate-like crystalline forms were visible, evenly distributed within the matrix. At the same time, the surface of the material appeared more compact and denser than in the case of samples containing laboratory PCM. No extensive cracks, delamination, or distinct separation of the phase change material from the geopolymer matrix were observed in any of the analyzed samples. This indicates that the composite preparation process proceeded correctly and that the PCM was effectively bonded to the matrix. Microscopic observations also confirm that the type of phase change material used influences the morphology and degree of development of the microstructure of geopolymer foams, which may be significant for their performance properties.

## 4. Discussion

The results confirm that the use of phase change materials simultaneously influences the development of the porous structure, mechanical properties, and thermophysical parameters of geopolymer foams. The thermal conductivity values λ obtained for all tested composites (0.0946–0.1088 W/m·K) fall within the range characteristic of lightweight geopolymer foams used as insulation materials [63,64]. In such materials, a relationship between density, porosity, and thermal conductivity is commonly observed—an increase in the pore fraction leads to a reduction in heat transport through the solid matrix of the material, resulting in a decrease in the λ value, though this is often at the expense of mechanical properties [63,64,65]. This trend is also evident in the conducted studies. The lowest thermal conductivity values were recorded for composites containing laboratory-grade PCM with particle sizes <1.6 mm and 2.0–2.5 mm, which simultaneously exhibited some of the highest thermal resistance values. In contrast, samples containing PX15 and PX20 materials exhibited higher λ values, which may indicate a more compact microstructure and a lower proportion of pores serving an insulating function.

The porosity analysis showed that the type of PCM used significantly influences the development of the porous structure of geopolymer foams. Particularly pronounced differences were observed between materials containing diatomite-paraffin granules and commercial PX materials. Diatomite has a well-developed capillary structure and a large specific surface area, allowing it to serve simultaneously as a PCM carrier and a component influencing the pore formation process during foaming [66,67,68]. In the tested composites, the highest porosity was obtained for the smallest diatomite-paraffin fraction, while the lowest was for the PX20 sample. This may indicate that the inorganic carrier used in PX materials acts partially as a microfiller, limiting the development of pore spaces. Similar observations have been reported for geopolymers containing porous PCM carriers and for diatomite-modified construction materials, where the developed structure of the carrier influenced the microstructure of the entire composite [66,68,69].

Despite significant differences in porosity, no clear correlation was observed between total porosity and compressive strength. Classical models describing the behavior of geopolymer foams indicate that an increase in porosity should lead to a decrease in mechanical properties due to a reduction in the continuity of the solid phase [63,70]. In the conducted studies, however, the highest strength was achieved by a sample containing PCM with a particle size of 1.6–1.8 mm, while some materials with increased porosity obtained higher strength values than the reference material. This means that not only the number of pores is crucial, but also their distribution, size, and the quality of the bond between the PCM particles and the geopolymer matrix. Similar phenomena were observed in geopolymers containing lightweight mineral fillers, where a homogeneous pore structure and good adhesion at the phase boundary led to improved mechanical properties despite an increase in total porosity [65,70,71].

Another significant finding of the study is the increase in the specific heat capacity of all PCM-containing composites compared to the reference material. The highest Cp values were recorded for the PX15 and PX20 samples, indicating their greatest potential for thermal energy storage. These results are consistent with the observations of other authors studying geopolymers and cementitious materials containing PCMs stabilized on mineral carriers [72,73,74]. It has been demonstrated that the presence of phase change materials increases the thermal inertia of building materials, enabling energy storage during temperature rise and its release during cooling [72,74,75]. Similar effects were obtained for geopolymer composites containing PCMs immobilized in cenospheres, porous kaolinite, and other mineral carriers [65,69].

An analysis of the λ and Cp values indicates that there is a characteristic trade-off between energy storage capacity and insulating properties. The PX15 and PX20 materials achieved the highest specific heat values, but at the same time were characterized by higher thermal conductivity. In contrast, composites containing diatomite-based PCM exhibited more favorable insulating properties at slightly lower Cp values. Similar relationships were observed in other PCMs intended for construction applications, where increasing the proportion of the active energy-storage phase led to improved thermal capacity at the cost of a partial increase in thermal conductivity [74,75,76]. The literature indicates that one possible direction for further optimization is the use of additives that increase the thermal conductivity of the PCM itself, such as graphite, graphene, or other carbon nanofillers [66,76,77].

Macroscopic observations and SEM analysis confirmed the effective binding of phase change materials to the geopolymer matrix. In samples containing laboratory-grade PCM, characteristic porous diatomite particles were visible, evenly distributed throughout the material’s structure. At the same time, no clear signs of paraffin leakage or extensive cracks at the phase boundary were observed. This indicates effective immobilization of the PCM within the carrier’s pores and good compatibility with the geopolymer matrix [66,68]. In the case of the PX-4, PX15, and PX20 samples, the microstructure was more homogeneous and compact, which is reflected in the results of porosimetry and thermal conductivity. Similar observations have been reported for geopolymer composites containing PCM stabilized in porous kaolinite and cenospheres, where the appropriate selection of the carrier influenced both the microstructure of the material and its performance properties [65,69].

A comprehensive analysis of the results indicates that both the laboratory-prepared diatomite- and paraffin-based PCM and commercial PX materials can be effectively used in lightweight geopolymer composites designed for thermal energy storage. Diatomite-paraffin materials exhibited a more favorable effect on the development of the porous structure and insulating properties, while PX materials provided higher specific heat values and greater energy storage potential. These results align with the current trend in the development of TES (Thermal Energy Storage) materials, which aims to achieve a balance between low thermal conductivity, adequate mechanical strength, and high thermal energy storage capacity [72,74,75,77].

## 5. Economic Analysis

An additional aspect analyzed in this study was the potential economic viability of using a laboratory-prepared phase change material based on diatomite and Flexol paraffin oil, compared to commercial PCMs from the Rubitherm PX series. The analysis was based on the actual purchase prices of the PX-4, PX-15, and PX-20 materials used in the study and the estimated costs of the raw materials used to produce the laboratory-prepared PCM. The calculations presented are indicative and refer to potential large-scale production, assuming the use of commonly available raw materials and standard manufacturing processes.

According to the purchase documentation, the average commercial price of Rubi-therm PX materials was approximately 20 EUR/kg, with prices for individual variants ranging from 20.02 to 20.41 EUR/kg. These materials are finished products containing paraffin-based PCM adsorbed onto a patented inorganic mineral carrier, characterized by high operational stability and repeatable thermal parameters. At the same time, their relatively high price may pose a significant limitation for large-scale implementations, particularly in the building materials sector, where the cost of a functional additive plays a key economic role.

In the case of the laboratory-grade PCM used in this study, the main components were diatomite and Flexol paraffin oil. Diatomite is a widely available natural mineral with a wholesale price of approximately 0.8 EUR/kg, while the price of Flexol paraffin oil, depending on the order size and degree of purification, is approximately 2.5 EUR/kg. The analysis was based on a composition of the laboratory PCMs consisting of approximately 60 wt.% paraffin oil and 40 wt.% diatomite, in accordance with the technology developed as part of this research. Based on these assumptions, the cost of the basic raw materials required to produce 1 kg of PCM composite can be estimated at approximately 1.8–2.5 EUR/kg.

The analysis also took into account the costs of the manufacturing process, which includes impregnating diatomite with paraffin, drying, granulation, and coating the granule surfaces with a thin stabilizing layer to limit their disintegration during transport and mixing with the geopolymer matrix. Under industrial conditions, this coating function can be fulfilled by inexpensive inorganic materials, such as soda-lime water glass, silica suspensions, or thin geopolymer layers, as well as modified industrial starches. The cost of the stabilizing material is low and typically does not exceed a few percent of the finished product’s value. Additionally, energy, labor, and granulation costs were taken into account, which were estimated at approximately 0.8–1.2 EUR/kg of finished material for large-scale production.

Taking all the assumptions into account, the total cost of producing 1 kg of laboratory-grade diatomite-paraffin granules can be estimated at approximately 2.5–4.0 EUR/kg. This means that the laboratory-developed PCMs can be about 5–8 times cheaper than commercial Rubitherm PX materials. From an economic standpoint, this represents a significant advantage, particularly in the large-scale production of insulation materials and geopolymer composites intended for energy-efficient construction.

It should be emphasized that the presented analysis is an estimate and does not include costs related to product certification, packaging, logistics, and the sales margin. The actual production cost may vary depending on raw material prices, production scale, and the technology used. Nevertheless, the results clearly indicate that the use of natural diatomite as a PCM carrier can significantly reduce the production cost of thermal energy storage materials while maintaining favorable performance characteristics. The PCMs developed in the laboratory, therefore, represent an attractive alternative to commercial solutions, both in economic terms and in terms of their potential for implementation in modern building materials.

The presented analysis focuses on the material’s capital costs (CAPEX) and allows for a comparison of the laboratory-developed PCM with commercial PX materials in terms of initial costs. From the perspective of practical construction applications, however, the costs incurred throughout the material’s entire service life (Life Cycle Cost Analysis, LCCA) are equally important. Although a detailed LCCA analysis is beyond the scope of this study and requires consideration of specific building parameters, local climatic conditions, energy prices, and the expected service life, it can be expected that the use of geopolymer foams containing PCM will help reduce the energy demand for heating and cooling buildings. This is due to the ability to store and gradually release thermal energy, which leads to a reduction in the amplitude of temperature fluctuations within the building envelope and a reduction in peak energy demand. Consequently, the higher initial cost associated with the use of PCM can be partially offset by lower operating costs over the entire service life of the building. Given that the laboratory-developed PCM has an estimated production cost approximately five to eight times lower than that of commercial PX materials, it can be expected that the total life-cycle cost of such a solution will also be more favorable. However, confirming this hypothesis requires conducting detailed LCCA analyses using actual building energy models, which is the focus of future research.

## 6. Limitations of the Study and the Stability of Laboratory-Prepared PCM

One of the key factors determining the practical usefulness of phase change materials is their long-term thermal stability during repeated melting and solidification cycles. Although this issue was beyond the scope of the present study, several observations can be made based on the results obtained and the available literature.

This study did not include tests involving multiple melting and solidification cycles of the laboratory-developed PCM; therefore, its long-term thermal stability was not directly evaluated. Nevertheless, macroscopic observations and SEM analysis revealed no signs of paraffin migration or damage to the granule structure following the production of geopolymer foams and the curing of the samples, indicating the effective immobilization of the PCM within the porous structure of diatomite. The obtained results are consistent with the literature, according to which diatomite, thanks to its well-developed capillary structure and high specific surface area, effectively retains liquid paraffin through capillary interactions and surface tension forces, thereby reducing the risk of leakage during phase transitions [66,68].

Studies by other authors have shown that diatomite-paraffin composites retain high thermal stability even after repeated melting and solidification cycles. Nassar et al. [66] demonstrated that appropriate modification of diatomite allows for further improvement of the composite’s dimensional stability and reduction in paraffin migration. Zhang et al. [69] found that porous geopolymer carriers effectively immobilize phase change materials, preserving their energy storage properties during repeated temperature changes. Similar conclusions were also presented by Gencel et al. [74] and El Majd et al. [72], who demonstrated that the use of inorganic carriers significantly improves the service life of shape-stabilized PCMs used in construction composites.

Despite the results obtained, it should be emphasized that a full assessment of the durability of the laboratory-developed PCM requires testing involving multiple thermal cycles in accordance with the relevant test procedures. Future work will include tests involving at least 500 melt-freeze cycles and a reanalysis of the material’s thermal, microstructural, and mechanical properties, which will allow for a comprehensive assessment of its long-term durability under operational conditions.

## 7. Prospects for the Durability of Geopolymer Composites Under Service Conditions

Given the planned use of geopolymer foams as thermal insulation materials for exterior building walls, their long-term durability under service conditions is a critical issue. In practice, materials of this type are exposed to fluctuating temperatures, freeze–thaw cycles, periodic moisture exposure, and UV radiation, which can lead to gradual degradation of the microstructure and deterioration of performance properties. The durability of composites is determined primarily by the stability of the geopolymer matrix, the integrity of the porous structure, and the behavior of the multiphase material during repeated phase transitions.

The results obtained in this study indicate that both the laboratory-prepared PCM based on diatomite and paraffin and the commercial PX materials were effectively integrated into the geopolymer matrix. SEM analysis and macroscopic observations revealed no signs of paraffin migration or localized damage to the interface between the PCM particles and the geopolymer binder. At the same time, the porous structure of diatomite promotes capillary retention of paraffin, limiting the risk of leakage during phase change, while the inorganic nature of the carrier ensures high thermal and chemical resistance.

It should be emphasized, however, that the actual durability of such composites should be evaluated taking into account the long-term effects of environmental factors. Of particular importance are tests involving repeated PCM melting and solidification cycles, freeze–thaw cycles, changes in humidity, and aging at elevated temperatures. These processes can lead to gradual degradation of the microstructure, the formation of microcracks, and changes in the material’s mechanical and thermal properties. For composites intended for outdoor use, it is also important to evaluate their resistance to carbonation, the effects of deicing salts, and repeated cycles of moisture sorption and desorption.

In light of the results obtained, it can be concluded that the developed geopolymer composites show promising potential for use as materials for passive thermal energy storage. At the same time, a comprehensive assessment of their service life requires further research conducted under conditions of long-term environmental aging, which will allow for the determination of changes in mechanical, microstructural, and thermophysical properties throughout the material’s service life.

## 8. Conclusions

This study analyzed the effect of various phase change materials on the properties of geopolymer foams produced using Class F fly ash. The study examined both a laboratory-developed PCM based on diatomite and Flexol paraffin oil with varying particle sizes, as well as the commercial materials Ru-bitherm PX-4, PX15, and PX20, used at a concentration of 7.5 wt.%. Based on the conducted research, the following conclusions were drawn:

1. The use of phase change materials significantly altered the microstructure and porosity of the geopolymer foams. The highest total porosity was obtained for the composite containing laboratory-prepared PCM with a particle size of <1.6 mm (20.88%), whereas the PX20 sample exhibited the lowest porosity (1.60%), indicating a strong influence of the PCM carrier on pore structure development.
2. All investigated composites exhibited low thermal conductivity, with λ values ranging from 0.095 to 0.109 W/m·K and thermal resistance values between 0.241 and 0.278 m^2^·K/W. The most favorable thermal insulation performance was obtained for the composite containing the 2.0–2.5 mm laboratory-prepared PCM fraction, which achieved the lowest thermal conductivity (0.095 W/m·K) and the highest thermal resistance (0.278 m^2^·K/W).
3. The addition of PCM increased the thermal energy storage capacity of the geopolymer foams. The specific heat increased from 1.494 kJ/kg·K for the reference sample to 1.778 kJ/kg·K for PX20, corresponding to an increase of approximately 19%. High values were also obtained for PX15 (1.740 kJ/kg·K).
4. The composite containing laboratory-prepared PCM with a particle size of 1.6–1.8 mm achieved the highest compressive strength (1.98 MPa), whereas the lowest value (1.02 MPa) was recorded for PX15. These results demonstrate that appropriate selection of the PCM type and particle size enables the preservation of satisfactory mechanical performance while improving thermal functionality.
5. Macroscopic observations and SEM analysis confirmed the homogeneous distribution of PCM within the geopolymer matrix and the absence of visible paraffin leakage or structural discontinuities. The laboratory-prepared diatomite–paraffin PCM produced a well-developed porous structure with total porosity reaching 20.88%, whereas the commercial PX20 material resulted in a much denser microstructure with a porosity of only 1.60%.
6. Commercial PX materials, particularly PX20, exhibited the highest specific heat (1.778 kJ/kg·K) but also the highest thermal conductivity (0.109 W/m·K). In contrast, the laboratory-prepared diatomite–paraffin PCM provided superior thermal insulation, reducing the thermal conductivity to 0.095 W/m·K while maintaining specific heat values between 1.512 and 1.552 kJ/kg·K, demonstrating a favorable balance between thermal insulation and heat storage capacity.
7. The economic analysis showed that the estimated production cost of the laboratory-prepared diatomite–paraffin PCM is approximately 2.5–4.0 EUR/kg, whereas the purchase price of commercial Rubitherm PX materials exceeds 20 EUR/kg. Consequently, the production cost may be reduced by approximately 80–85% while maintaining comparable thermophysical performance, indicating considerable potential for large-scale building applications.

The results confirm that both laboratory-grade and commercial PCMs can be effectively used as functional additives in geopolymer foams intended for energy-efficient construction. The use of diatomite as a low-cost PCM carrier, enabling simultaneous improvement of thermal insulation properties and thermal energy storage capacity, appears to be a particularly promising solution. Further research should focus on evaluating the durability of materials during repeated heating and cooling cycles, analyzing the long-term stability of PCM in a geopolymer matrix, and determining the energy efficiency of composites under real-world building conditions.

## Figures and Tables

**Table 1:** The thermophysical properties of commercial and laboratory-prepared phase change materials used in the study.

Parameter	Diatomite + Flexol	PX-4 (RT-4)	PX15 (RT15)	PX20 (RT20)
Transition temperature [°C]	−30 to −3	−7 to −3	10–17	16–20
λ [W/m·K]	0.106–0.121	0.20	0.20	0.20
Cp [kJ/kg·K]	1.16–1.55	2.0	2.0	2.0
Flash point [°C]	260	>90	150	150
PCM content [wt.%]	69.7	~60	~60	~60
PCM type	Flexol paraffin	RT-4 paraffin	RT15 paraffin	RT20 paraffin
Media type	diatomite	inorganic porous carrier	inorganic porous carrier	inorganic porous carrier

**Table 2:** Composition and designations of the geopolymer composites under investigation.

Sample ID	Sand, g	Microspheres, g	Fly Ash, g	Cement, g	Stabilizer, g	PCM, g	H_2_O_2_, mL	Activator, mL
AAM-REF	80	160	795	100	5	–	25	350
<1.6 mm_7.5 wt.%	80	160	795	100	5	86	25	370
1.6–1.8 mm_7.5 wt.%	80	160	795	100	5	86	25	370
1.8–2.0 mm_7.5 wt.%	80	160	795	100	5	86	25	390
2.0–2.5 mm_7.5 wt.%	80	160	795	100	5	86	25	400
>2.5 mm_7.5 wt.%	80	160	795	100	5	86	25	400
PX-4_7.5 wt.%	80	160	795	100	5	86	25	400
PX15_7.5 wt.%	80	160	795	100	5	86	25	400
PX20_7.5 wt.%	80	160	795	100	5	86	25	400

**Table 3:** The porosity of geopolymer composites containing PCM.

Material	Total Porosity, %	Pore Diameter Range, μm	Total Surface Area, m^2^/g	Total Intruded Volume, cm^3^/g
AAM-REF	8.37	4.26–239.51	0.025	0.310
<1.6 mm_7.5 wt.%	20.88	4.27–230.68	0.062	0.522
1.6–1.8 mm_7.5 wt.%	13.40	4.27–230.68	0.047	0.394
1.8–2.0 mm_7.5 wt.%	12.83	4.26–236.21	0.051	0.367
2.0–2.5 mm_7.5 wt.%	6.93	4.26–236.21	0.048	0.315
>2.5 mm_7.5 wt.%	15.65	4.26–239.51	0.080	0.522
PX-4_7.5 wt.%	11.16	4.26–167.77	0.033	0.360
PX15_7.5 wt.%	7.17	4.26–167.77	0.016	0.231
PX20_7.5 wt.%	1.60	4.27–228.42	0.011	0.084

**Table 4:** Compressive strength of geopolymer composites containing PCM.

Material	Compressive Strength, MPa
AAM-REF	1.12
<1.6 mm_7.5 wt.%	1.64
1.6–1.8 mm_7.5 wt.%	1.98
1.8–2.0 mm_7.5 wt.%	1.34
2.0–2.5 mm_7.5 wt.%	1.08
>2.5 mm_7.5 wt.%	1.74
PX-4_7.5 wt.%	1.62
PX15_7.5 wt.%	1.02
PX20_7.5 wt.%	1.60

**Table 5:** Thermal properties of geopolymer composites containing PCM.

Material	Density, kg/m^3^	λ at 0–20 °C, W/m × K	R at 0–20 °C, m^2^ × K/W	Cp at 27.5–32.5 °C, kJ/kg × K
AAM-REF	402.62	0.103	0.259	1.494
<1.6 mm_7.5 wt.%	367.22	0.095	0.277	1.512
1.6–1.8 mm_7.5 wt.%	426.43	0.104	0.257	1.526
1.8–2.0 mm_7.5 wt.%	450.94	0.101	0.264	1.513
2.0–2.5 mm_7.5 wt.%	353.85	0.095	0.278	1.532
>2.5 mm_7.5 wt.%	394.74	0.099	0.266	1.552
PX-4_7.5 wt.%	398.16	0.098	0.275	1.638
PX15_7.5 wt.%	394.20	0.105	0.249	1.740
PX20_7.5 wt.%	397.97	0.109	0.241	1.778

## Data Availability

The original contributions presented in this study are included in the article. Further inquiries can be directed to the author.
